# Identification and association of the single nucleotide polymorphisms, C−509T, C+466T and T+869C, of the TGF-β1 gene in patients with asthma and their influence on the mRNA expression level of TGF-β1

**DOI:** 10.3892/ijmm.2014.1894

**Published:** 2014-08-11

**Authors:** MICHAŁ PANEK, TADEUSZ PIETRAS, ARTUR FABIJAN, JAN ZIOŁO, ŁUKASZ WIETESKA, BEATA MAŁACHOWSKA, WOJCIECH FENDLER, JANUSZ SZEMRAJ, PIOTR KUNA

**Affiliations:** 1 Department of Internal Medicine, Asthma and Allergy, Medical University of Lodz, 90-153 Lodz, Poland; 2 Department of Pneumology and Allergology, Medical University of Lodz, 90-153 Lodz, Poland; 3 Students Research Group, Department of Internal Medicine, Asthma and Allergy, Medical University of Lodz, 90-153 Lodz, Poland; 4 Department of Medical Biochemistry, Medical University of Lodz, 92-215 Lodz, Poland; 5 Department of Pediatrics, Oncology, Hematology and Diabetology, Medical University of Lodz, 91-738 Lodz, Poland

**Keywords:** asthma, inflammation, transforming growth factor-β1 expression, transforming growth factor-β1 polymorphisms, single nucleotide polymorphism, genetics

## Abstract

Transforming growth factor-β1 (TGF-β1) is an important fibrogenic and immunomodulatory cytokine participating in the pathogenesis of a number of illnesses related to the growth, differentiation and migration of cells. It also plays a key role in inflammation, atherosclerosis, vascular inflammation and asthma. The aim of the present study was to evaluate the association between the expression of the TGF-β1 gene and its genetic polymorphisms, and the disease phenotype. The study comprised 173 patients with asthma, as well as 163 healthy volunteers as a control group. The gender profiles of the groups were similar (p=0.8415). Genotyping was performed by polymerase chain reaction (PCR)-high resolution melting (HRM). The results were verified by sequencing. Gene expression was evaluated by RT-PCR. This study evaluated the role and frequency of genetic polymorphisms (C−509T, C+466T and T+869C) of the TGF-β1 gene in the study group (patients with asthma) and the control group (healthy volunteers). The results obtained for the patients and healthy controls were as follows: C−509T single nucleotide polymorphism (SNP) (controls, TT/CT/CC-0.4444/0.5309/0.0247; patients, TT/CT/CC-0.3699/0.6012/0.0289), C+466T SNP (controls, TT/CT/CC-1.000/0.000/0.000; patients, TT/CT/CC-1.000/0.000/0.000) and T+869C SNP (controls, TT/CT/CC-1.000/0.000/0.000; patients, TT/CT/CC-1.000/0.000/0.000). Only the C−509T polymorphism was found to play a significant role in the pathogenesis of asthma, as well as a risk factor in the loss of the clinical control of the disease [TT vs. CC/CT, odds ratio (OR) 2.38; confidence interval (CI) 1.22–4.66; p=0.0103]. A significant difference was noted between the study and control groups with regard to the mRNA expression of TGF-β1 (p=0.0133). A higher level of expression of the TGF-β1 gene correlated with the time of diagnosis of patients over 16 years of age (p=0.0255). This study demonstrates that the C−509T SNP is a significant clinical risk factor for asthma and that the TGF-β1 cytokine contributes to the progression of the illness.

## Introduction

The genetic determination of the development of asthma is based on a multifactorial inheritance model of the illness involving approximately 100 genes (1); however, only 50% of these genes have been confirmed in tests (1,2). Current research interest is focused on the genetic markers which may correlate with many asthma endotypes: parameters from pulmonary function tests, bronchial hyperresponsiveness, as well as environmental and inflammatory factors (1,3). Current views on the pathogenesis of asthma indicate that a key role is played by over 100 mediators of inflammation, as well as complex structural changes in the bronchial wall (1,4,5).

The main role of Th2 lymphocytes and interleukin (IL)-4, IL-9 and IL-13 in the development of asthma is the activation of a number of inflammatory cells, the release of a number of pro-inflammatory mediators and the hypersecretion of mucus (1,6). IL-5, IL-6 and transforming growth factor-β (TGF-β) are responsible for bronchial hyperresponsiveness and the development of structural changes occurring in the bronchial wall (6–8). The key role of TGF-β as a growth factor responsible for the prolongation of the repair and activation processes of microfibroblasts has been noted in several studies regarding inflammation of the respiratory tract and its remodeling during asthma (1,8–10). TGF-β belongs to a superfamily comprising over 30 mammalial cytokines including, *inter alia*, various isoforms of the same transforming factor, TGF-β (9–11).

TGF-β isoforms 1, 2 and 3 are located on chromosomes 19q13, 1q41 and 14q24, respectively (12). In humans, this agent is found in 3 inactive positions connected with the latent associated protein (LAP), as well as latent TGF-β-binding protein (LTBP) (13,14). TGF-β1 is found in endothelial and hematopoietic cells, as well as connective tissue; TGF-β2 is found in endothelial tissue and neurons; TGF-β3 is found in connective tissue cells. The multi-faceted actions of TGF-β are realised through a number of receptors, I TGF-β, II TGF-β and III TGF-β, through participation in the specific Smad protein [mothers against decapentaplegic (MAD) and SMA gene; whose names are derived from the blend of 2 homologous Sma proteins, as well as MAD occurring as a response to *Caenorhabditis elegans* and *Drosophila melanogaster*] (13–17). Smads are responsible for the transmission of extracellular signals from TGF-β ligands to the nucleus, where the downstream activation of TGF-β gene transcription takes place (15–20). The regulation of TGF-β mRNA transcription is based on a number of factors, including nerve growth factor, operating through the Erg-1 site and oncogenes (*src*, *abl*, *ras*, *jun* and *fos*) (9,21–24). TGF-β promoters display little homology and comprise various elements responsible for the independent regulation of gene expression. Unlike the TGF-β2 and TGF-β3 genes, the promoter region of TGF-β1 does not include TATA and CAAT boxes (9,21).

To date, over a hundred single nucleotide polymorphisms (SNPs) have been identified, as well as 11 other types of genetic variations for TGF-β1 (25), which may exert an influence on the regulation of gene expression, and may be connected with such respiratory tract illnesses as asthma (26). The C−509T polymorphism (rs1800469) of the TGF-β1 promoter has been found to be related to an elevated plasma level of TGF-β1, an elevated level of total IgE and an increased risk of remodeling bronchi, as well as the development of asthma (26–31). The T+869C (rs1800470) SNP may play a significant role in the level of the mRNA expression of TGF-β1 (26).

The role of the C+466T (rs200482214) polymorphism in the pathogenesis of inflammation in the course of asthma has not yet been clearly identified. However, the presence of the residue change R [Arg] → C [Cys] allows its influence on the level of TGF-β1 mRNA expression to be identified (34).

However, it is necessary to emphasise that significantly large discrepancies exist in the frequency of the occurrence of SNPs (C−509T and T+869C) of the TGF-β1 gene based on ethnic and geographical variation, for example Europe vs. Asia. In turn, previous studies are not coherent as regards the role of SNPs in the etiopathogenesis of asthma and their influence on the risk of developing the illness (26,32,33). Similarly, no frequency data exist for C+466T (34).

Thus, the aim of this study was to evaluate the frequency of the occurrence of the C−509T, C+466T and T+869C SNPs of the TGF-β1 gene in a European population from Poland, and to identify the existence of any correlation between the frequency of these SNPs and the risk of developing asthma. Particular emphasis was paid to the association between the occurrence of the tested genotypes and the level of the mRNA expression of TGF-β1.

## Materials and methods

This study was approved by the local Ethics Committee (Consent of Research Review Board at the Medical University of Lodz, Poland; no. RNN/133/09/KE and no. RNN/31/14/KE). At the commencement of the study, participants were invited to attend voluntarily. Prior to enrollment, written informed consent was obtained from each participant.

The present study was conducted on a group of 173 patients with bronchial asthma. The diagnosis of asthma was established according to the Global Initiative for Asthma (GINA) recommendations, based on clinical asthma symptoms and a pulmonary function test. The level of severity of asthma and control was determined on the basis of the GINA study guidelines.

The patients provided a medical history comprising, *inter alia* gender, obesity, tobacco smoking and the duration of bronchial asthma, as well as allergies to house dust mites, animal fur, mold spores, cockroach allergens and hypersensitivity to non-steroidal anti-inflammatory drugs (NSAIDs). These statistics were used to determine their role in the development of resistance to glucocorticoids, as well as to establish whether they were primary or secondary to the genetic factors. Objective examinations were also performed.

The results of pulmonary function and allergological tests were obtained from the individual medical records of the patients. If these were unavailable, the relevant examinations were performed during the recruitment visit.

The exclusion criteria were as follows: the presence of clinically significant exacerbations; the use of drugs, such as rifampicin, phenobarbital, phenytoin or ephedrine which may induce resistance to glucocorticoids; signs of viral generalised or respiratory tract infections; failure to comply with the doctor’s recommendations.

The control arm included a group of 163 healthy adults who met the following criteria: no history or symptoms of either bronchial asthma or other pulmonary diseases; no history or symptoms of allergy; no history or symptoms of atopic dermatitis; no history or signs of hypersensitivity to aspirin; negative results of skin tests for 12 common allergens; no first-degree relatives with bronchial asthma or atopic disorders, as previously described (35–38). Healthy volunteers were selected for the tests from the general population. The selection was random.

According to the standards of the Polish Society for Pulmonary Diseases, the analysis of obstructive disorders and disease severity was based on the best of 3 spirometry readings. The correlation analysis took into consideration forced expiratory volume in 1 sec (FEV1) expressed in liters, FEV1% (A/N% - percentage ratio of the measured to expected value) expressed as percentage of the expected value and the FEV1% forced vital capacity (FVC) index (FEV1 to FVC ratio), expressed as absolute values. Spirometry tests were conducted in the Outpatient Department according to the standards of the European Respiratory Society (ERS)/American Thoracic Society (ATS), while allergological tests were performed according to the guidelines of the European Academy of Allergy and Clinical Immunology (EAACI), as previously described (35–38).

The level of asthma control was assessed using the Asthma Control Test (ACT™), which is clear and easy for patients, and consists of 5 questions. It was developed by Nathan *et al* (39). Bronchial asthma control was calculated based on the following ACT scores: 0 to 19 points, no asthma control; 20 to 24 points, partially controlled asthma; 25 points, well-controlled asthma, as previously described (35–38).

The study included 336 participants: 163 healthy subjects and 173 patients with asthma. The gender proportion within the groups was similar: there were 63.58% females in the asthma group vs. 62.58% in the control group, p=0.8415. Detailed profiles of the 2 groups are presented in Tables I and II.

Venous blood samples were collected from the participants into EDTAK3 vacuum blood collection tubes (SARSTEDT AG & Co.; Nümbrecht, Germany). DNA was obtained from the peripheral blood leukocyte fraction. The genetic material was isolated using the QIAamp DNA Blood Mini kit (Qiagen Inc., Valencia, CA, USA) according to the guidelines provided by the manufacturer.

The identification of the SNPs of the TGF-β1 gene was conducted using the polymerase chain reaction (PCR)-high-resolution melting (HRM) technique. The exponential amplification of the C−509T, C+466T and T+869C polymorphism DNA segments was carried out using a forward and reverse primer according to the standard PCR protocol. The detailed primer sequences are presented in Table III. Primer binding to complementary DNA matrix sites was conducted according to the temperatures presented in Table III.

The amplified PCR product was diluted 50 times to obtain a matrix (1:50). The first stage of HRM analysis involved the amplification of the investigated DNA fragment containing the analysed C−509T, C+466T and T+869C SNPs on the 1:50 matrix using a forward and reverse primer. The primer sequences are presented in Table IV. This was followed by denaturation and slow renaturation to form a heteroduplex.

At the final stage, the mixture was subjected to precise denaturation in the presence of an intercalating stain. The C−509T, C+466T and T+869C SNP DNA fragments were identified by melting curve analysis using the LightScanner^®^ High Sensitivity Master Mix (Idaho Technology Inc., Salt Lake City, UT, USA). The obtained product was subjected to internal control using a molecular probe phosphorylated at the 3′-terminal portion (unlabeled, 3′ blocked oligonucleotide: 5′-GAC CCT TCC ATC CCT CAG GTG TCC TG-Pho-3′ for C−509T SNP; 5′-TTG AGC CTC AGC AGA CGC AGC TCT GCC C-Pho-3′ for C+466T SNP; and 5′-GCT GCG GCT GCT GCC GCT GCT GCT-Pho-3′ for T+869C SNP).

HRM analysis was then performed using the LightScanner Master Mix (Idaho Technology Inc.) and the selected SNP samples were then sequenced to confirm the presence of the appropriate PCR-HRM reaction product and its polymorphism.

**Figure 1:**
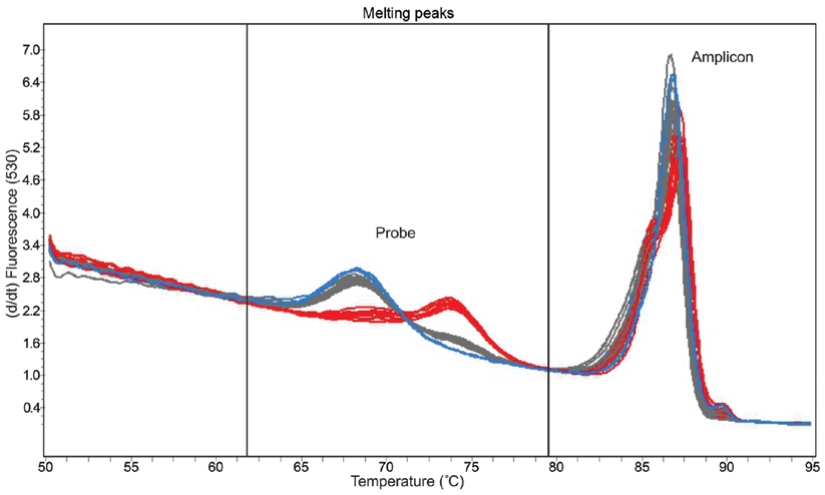
The melting curves obtained for the C−509T single nucleotide polymorphism (SNP) of transforming growth factor-β1 (TGF-β1) over the entire temperature range. Probe-target melting was observed between 62–79°C, while the amplicon melting occurred between 80–90°C.

Fig. 1 shows the probe normalization process with comparison of the denaturation curves and automatic identification of genotypes on the basis of differences in the melting temperature (CC and TT homozygotes) and denaturation curve shapes (CT heterozygotes) for the C−509T polymorphism of TGF-β1.

**Figure 2:**
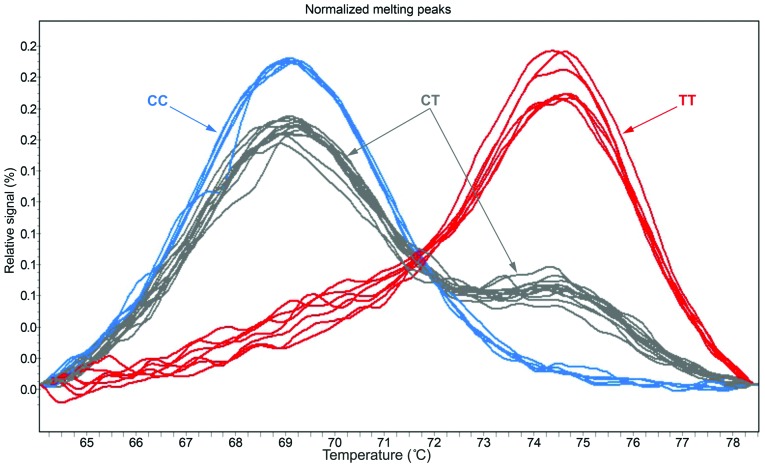
The normalised and temperature-shifted melting curves for the C−509T polymorphism. The melting curves depict homozygous (CC), heterozygous (CT) and wild-type (TT) mutations.

**Figure 3:**
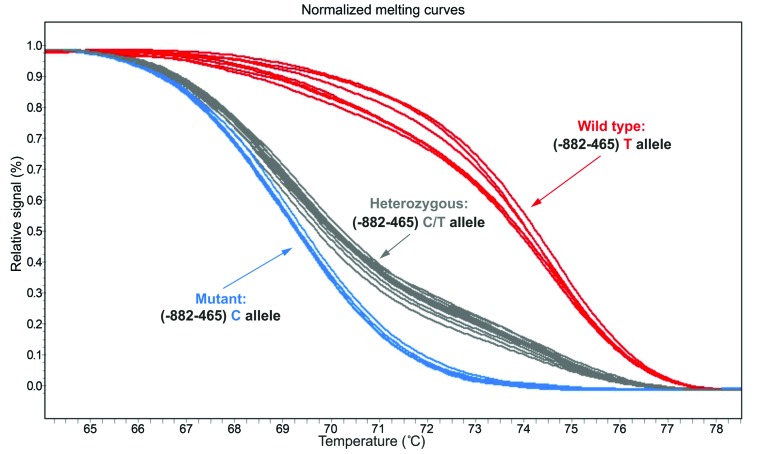
Normalised and temperature-shifted melting curves for the C−509T polymorphism of transforming growth factor-β1 (TGF-β1). The melting curves depict homozygous (CC), heterozygous (CT) and wild-type (TT) genotypes.

**Figure 4:**
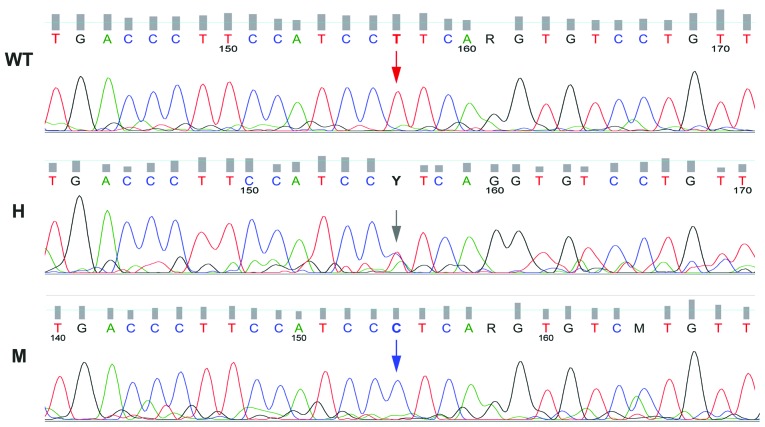
Detailed sequencing results of the C−509T single nucleotide polymorphism (SNP) with the polymorphic form indicated (arrow): WT, wild-type; H, heterozygous; M, mutation.

Figs. 2 and 3 show the amplicon normalization process and the comparison of the denaturation curves and automatic identification of genotypes based on differences in the melting temperature (CC and TT homozygotes) and denaturation curve shapes (CT heterozygotes) for the C−509T polymorphism. Examples sequences of C−509T SNP are presented in Fig. 4.

An amount of 10 μg total RNA was extracted from the peripheral blood lymphocytes using TRI Reagent^®^ Solution (Ambion, Grand Island, NY, USA) according to the standard acid-guanidinium-phenol-chloroform method [Chomczynski and Sacchi (40)]. The extracted RNA was analysed by agarose gel electrophoresis and only cases with preserved 28S, 18S and 5S ribosomal RNA bands, indicating good RNA quality, were used in this study. Total RNA was digested using DNase (Gibco, Carlsbad, CA, USA) at room temperature for 15 min. The amount of purified RNA was determined using spectrophotometry at 260 nm with a NanoDrop Analyser (ND-100; Nanodrop Technologies, Wilmington, DE, USA). The purity was verified according to the 260/280 nm ratio, with values between 1.8 and 2.1 indicating that RNA quality was optimal and suitable for quantitative reverse transcription PCR (RT-qPCR) (35,40).

The reverse transcription of 1 μg RNA was performed using an AccuScript PfuUltra II RT-PCR kit (Agilent Technologies, Santa Clara, CA, USA). The cDNA was subjected to quantitative PCR using gene-specific primers (5′-GGT ACC TGA ACC CGT GTT GCT-3′ and 5′-TGT TGC TGT ATT TCT GGT ACA GCT C-3′) (Sigma-Aldrich, Steinheim, Germany) for TGF-β1 and glyceraldehyde 3-phosphate dehydrogenase (GAPDH) [5′-AGC CAC ATC GCT CAG ACA-3′ and 5′-GCC CAA TAC GAC CAA ATC C-3′; Institute of Biochemistry and Biophysics, Polish Academy of Sciences (IBB PAS), Warsaw, Poland] using a Brilliant II SYBR-Green qRT-PCR Master Mix kit (Stratagene, La Jolla, CA, USA). Amplification was performed using the normal 2 steps and a standard thermal profile. Primer annealing temperature was 61°C, and primer annealing time was 20 sec. An Agilent Technologies Stratagene Mx3000P was used for the PCR reaction. For each sample, the threshold cycle (C_T_) values were calculated with the help of Mx-Pro software. The RT-PCR amplification of the TGF-β1 gene was compared to that of GAPDH, a house-keeping reference gene, and ΔC_T_ values were determined (ΔC_T_ = C_T,GENE_ - C_T,GAPDH_). Real-time PCR data was automatically calculated with the data analysis module. The results were analysed according to the 2^−ΔΔCT^ method, as previously described (35,41,42). Validation of PCR efficiency was performed using a standard curve, as previously described (35).

The level of significance was set at p=0.05. Statistical analysis was performed using STATISTICA version 10 (2011, licence no. AXAP202E504303AR-A; StatSoft, Inc., Tulsa, OK, USA). The genotyping was performed by 2 investigators who were unaware of the phenotypes. Hardy-Weinberg equilibrium (HWE) test of SNP was performed using Michael H. Court’s (2005–2008) online calculator (http://www.tufts.edu/~mcourt01/Documents/Court%20lab%20-%20HW%20calculator.xls).

## Results

The frequency distributions of the tested TGF-β1 polymorphisms were evaluated. The frequencies of the homozygous (TT and CC) and heterozygous (CT) C−509T SNP are presented in Table V. The T allele was found to occur in 71% and the C allele in 29% of the control group, while the T allele and C allele were found in 67 and 33% of the asthma patient groups, respectively.

Genotyping of the C+466T and T+869C SNPs did not reveal the presence of mutated homozygotes or heterozygotes. Only wild-type homozygotes were found (WT). The test results are presented in Tables VI and VII.

As the C+466T and T+869C SNPs were unproven, only the C−509T SNP was included in further analyses. For the C−509T polymorphism, the genotype distribution was evaluated based on the level of severity of the course of asthma, and the level of the clinical control of symptoms was tested depending on the severity of the illness. The results are presented in Tables VIII and IX.

The influence of the C−509T polymorphism on the degree of control of clinical asthma symptoms was evaluated, which may significantly influence the phenotype of the patient. The comparative analysis is shown in Table X.

**Figure 5:**
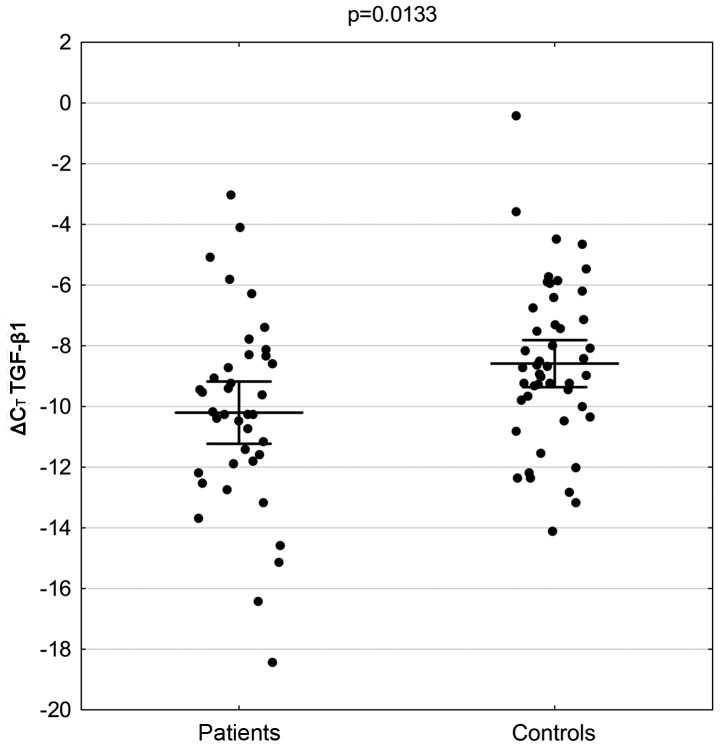
The level of expression of transforming growth factor-β1 (TGF-β1) in the patient and control groups. ---, mean; **I**, mean ± 0.95 level of confidence; ●, source data.

Differences in the expression of TGF-β1 mRNA between the analysed populations are presented in Table XI and Fig. 5. The association between the level of expression of TGF-β1 and the severity of the course of asthma is presented in Table XII.

**Figure 6:**
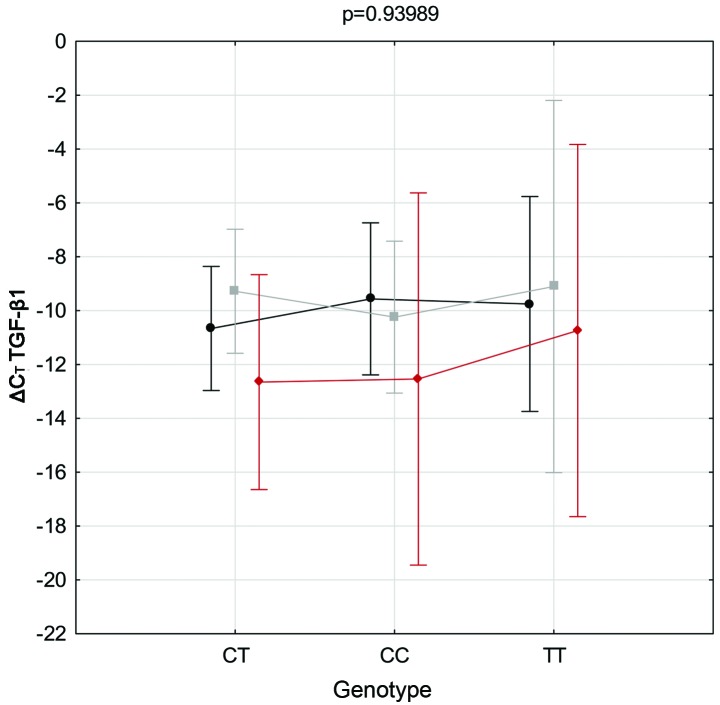
Correlation between C−509T genotype on the level of expression of transforming growth factor-β1 (TGF-β1) mRNA and the level of asthma severity. Asthma severity: black, severe; grey, moderate; red, mild.

**Figure 7:**
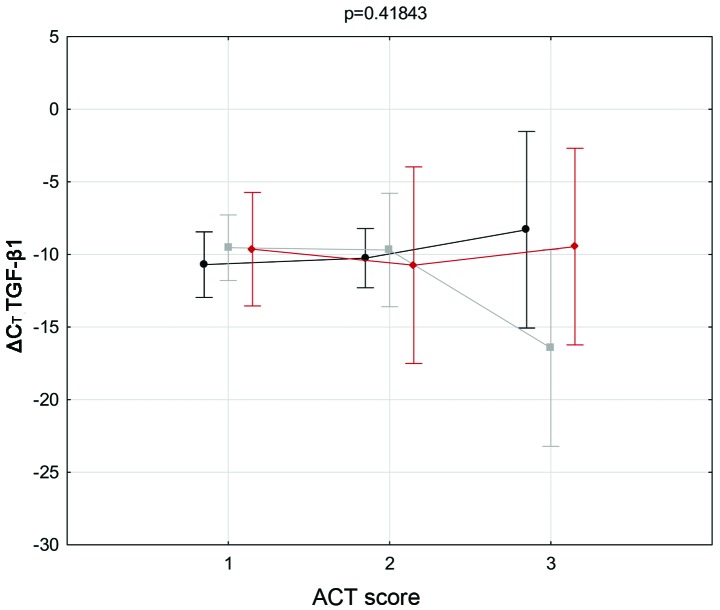
Correlation between the degree of asthma control and the C−509T polymorphism, and the level of expression of transforming growth factor-β1 (TGF-β1) mRNA. C−509T polymorphism genotypes: black, CT; grey, CC; red, TT. Asthma control test (ACT): <20 points, 1; 20–24 points, 2; 25 points, 3.

The influence of the genotype of the C−509T polymorphism on the mRNA expression level of TGF-β1 is shown in Fig. 6. The association between the TGF-β1 mRNA expression and asthma symptom control according to ACT is shown in Fig. 7.

In addition, we tested the association between the C−509T SNP and spirometric parameters (FEV1 in liters, p=0.672; FVC in liters, p=0.741; FEV1% FVC as a percentage, p=0.588). No significant correlation was found. The association between TGF-β1 mRNA expression and a number of other factors was also investigated. Other factors incuded the presence of allergies (seasonal allergies, year-round allergies, p=0.454), doses of glycocorticosteroids taken (inhaled and systemic, p=0.084), occasional smoking (p=0.679), as well as the number of cigarettes smoked per day (p=0.988). No significant association was observed between the number of asthma attacks per year and the level of TGF-β1 mRNA expression (p=0.283).

**Figure 8:**
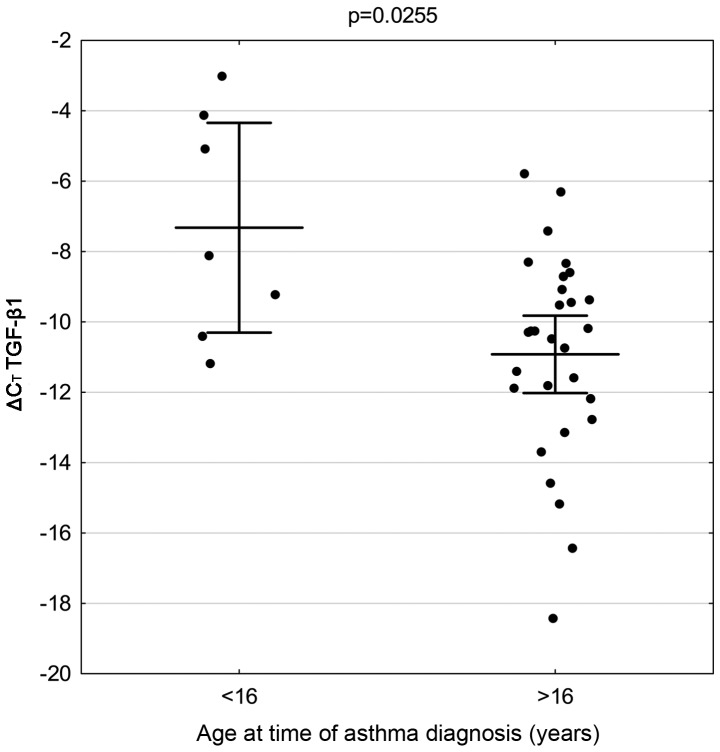
Correlation between transforming growth factor-β1 (TGF-β1) mRNA expression and the duration of asthma, with the age at which the condition developed. The analysis was carried out on the asthma group. ---, mean; **I**, mean ± 0.95 level of confidence; ●, source data.

The association between the mRNA expression level of TGF-β1 and the duration of asthma from the time of diagnosis was also investigated. Much higher levels of TGF-β1 expression were observed in patients who developed asthma after the age of 16, as well as in those had been ill for longer. The results of the analysis are presented in Fig. 8.

## Discussion

Asthma is a heterogenous disease. Its clinical picture is a result of interactions between different environmental factors and numerous genetic determinants (1,36–38). A complex etiopathogenetic process leading to the development of inflammation, bronchial hyperreactivity, recurrent episodes of wheezing and dyspnea is determined by interactions between genetic and environmental factors. The clinical picture results from complex gene-gene and gene-environment interactions (1,36–38). It has been estimated that approximately 100 genes are involved in the etiopathogenesis of asthma. However, the genetic constituent of the variance has not yet been ultimately estimated, and a number of genes have unclear biological significance and unknown mechanisms of action (1,3,36–38).

This study evaluated the frequency and the role of TGF-β1 gene polymorphisms (C−509T, C+466T and T+869C) in a group of patients suffering from asthma and a control group of healthy subjects. The following results were obtained for the patients and controls: C−509T SNP (controls, TT/CT/CC-0.4444/0.5309/0.0247; patients, TT/CT/CC-0.3699/0.6012/0.0289), C+466T SNP (controls, TT/CT/CC-1.000/0.000/0.000; patients, TT/CT/CC-1.000/0.000/0.000) and T+869C SNP (controls, TT/CT/CC-1.000/0.000/0.000; patients, TT/CT/CC-1.000/0.000/0.000). The populations studied were hence not in equilibrium, which can be explained by a lethal character of changes in nucleotide variances of the gene analysed. Another potential reason for the imbalance of the genotype distribution may be associated with the matching of sexual partners not always being random. If the partner is selected (unconsciously) on the basis of certain features, the genotype distribution in the population is far from the equilibrium. A statistically significant correlation between the group of patients with asthma and the controls was shown only for the TGF-β1 gene C−509T polymorphism, whereas for the 2 remaining polymorphisms, C+466T SNP and T+869C, no significant correlation was observed. The study on the C+466T and T+869C polymorphisms did not reveal the presence of allelic variants in the population of examined healthy subjects and asthmatic patients. The results were verified and confirmed by sequencing. Hence, it may be hypothesised that the C+466T and T+869C polymorphisms of the TGF-β1 gene occur very rarely and a mutation rather than an SNP should be considered. Obviously, confirmation of this fact requires a larger sample of individuals and multicenter analyses.

The C−509T polymorphism of the TGF-β1 gene indicates a significant association with the level of severity of asthma symptoms measured by the asthma control test ACT. A higher frequency of the TT genotype of the C−509T SNP was noted in the group of patients with an uncontrolled phenotype of asthma symptoms. In the control group, a lower frequency of the TT genotype and a higher one of the CC genotype was identified for the polymorphism studied. The T allele of the C−509T SNP of TGF-β1 appeared to be a risk allele for the loss of the clinical control of the disease. The product of the expression of 2 TT alleles of the SNP was found to be a determinant which most strongly correlated with the loss of asthma symptom control [TT vs. CC/CT odds ratio (OR) 2.38; confidence interval (CI) 1.22–4.66; p=0.0103]. Thus, it was demonstrated that the TGF-β1 gene C−509T polymorphism plays an important role in the pathogenesis of uncontrolled asthma. The allele frequencies in the present study were as follows: 71% for the T allele, 29% for the C allele in the healthy subjects; 67% for the T allele and 33% for the C allele in the group of patients. These outcomes are similar to those observed by other authors who analysed the role of the discussed polymorphism in the pathogenesis of asthma (28,31,43). The extreme results of the C−509T SNP (the C allele frequency being as high as 100%), determined in some investigations, may raise doubts as to the methodology used in these studies. It should be however emphasised that the data derived from the National Center for Biotechnology Information (NCBI) dbSNP [reference SNP (refSNP) Cluster report: rs 1800469] concerns geographically and ethnically differentiated populations (the T allele frequency from 14 to 67%; the TT genotype frequency from 0 to 56%; the CC frequency from 20 to 100%). These are the results for the frequencies of alleles and genotypes of the C−509T polymorphism recognised in different disease entities. The aim of their presentation was to depict the spectrum of the nucleotide variance distribution of the SNP as compared to other scientific studies.

Among numerous identified SNPs in TGF, the C−509T polymorphism seems to be a commonly recognised risk factor for the development of asthma and atopy. It also affects the mRNA level of TGF-β1 (43). The C−509T SNP correlates with wheezing illness in infants, a severe course of asthma and elevated IgE levels in serum, which has been confirmed by certain studies (27–29,32,43). It should be noted that, to the best of our knowledge, no such detailed studies on the role of C−509T, C+466T and T+869C polymorphisms of TGF-β1 have been carried out to date among the Polish population of asthmatic patients, using the novel methods presented in this study. In sum, out of all the examined SNPs, only one, C−509T, appeared to be a clinically significant haplotype and a risk factor for asthma (the TT genotype).

The present study indicated different levels of TGF-β1 mRNA expression between the populations analysed (p=0.0133). A higher TGF-β1 blood level was detected in the group of patients as compared to the controls. There was no association observed between the TGF-β1 mRNA expression and the severity of asthma (p=0.2772). Although the groups significantly differed in the TGF-β1 blood level, the mRNA level did not affect the degree of asthma symptom control. The multivariate analysis of the association between the TGF-β1 C−509T polymorphism, the gene expression level (C_T_) and the degree of asthma severity (Fig. 6) did not demonstrate any correlation of the SNP with TGF-β1 blood levels (p=0.9398). Moreover, the association between the level of asthma control assessed by using the ACT with ΔC_T_ TGF-β1 mRNA and the C−509T haplotype of TGF-β1 were analysed. Although statistically significant correlations (p=0.4184) were not found, the TT genotype showed a tendency towards a higher gene expression level and thus may be considered responsible for the loss of asthma control (Fig. 7). Obviously, further studies on a greater number of patients are required to confirm these observations. A statistically significant correlation between ΔC_T_ TGF-β1 mRNA and the duration of the disease was observed (Fig. 8). A higher TGF-β1 expression level correlated with the time of the disease diagnosis in patients above 16 years of age (p=0.0255). This may be explained by a longer duratino of TGF-β1 impact on the processes of growth, differentiation and cell migration in the bronchi of patients with asthma, as well as its regulatory impact on the response of the inflammatorily changed cells in the airways. These are the processes of formation and degradation of extracellular matrix components, chemotaxy and bronchial epithelial cell apoptosis (44–47).

Moreover, the association between SNPs and the parameters from pulmonary function tests (FEV1, FVC, FEV1% FVC) was not displayed in the present study. No correlation between ΔC_T_ TGF-β1 mRNA and the presence of allergy (lack of allergy, seasonal allergy and all-season allergy), the number of asthma exacerbations, the dose of inhaled glycocorticosteroids and smoking was shown either. The analysis of the factors affecting the level of the TGF-β1 mRNA expression did not reveal any statistically significant correlations. Particular attention should be paid to the fact that the groups studied were too small to achieve the established p-value. Furthermore, there are certain studies which confirm the present observations as regards this matter (32,33). External factors differentially affect the expression of 5 TGF-β isoforms, which in *in vitro* conditions bind together and activate the same TGF-β receptors and similar signalling pathways, as well as exert similar effects of action (44–48). Thus, the used methods of post hoc analysis have some limitations as regards the evaluation of the selected factors and their impact on certain elements of complex signalling pathways, in which TGF-β1, among others, is involved (30,47–51). Moreover, the selective evaluation of the role of external determinants on the level of TGF-β1 expression due to its individuality does not consider complex interactions initiating the TGF-β1 binding to specific TGF-β1 receptors. Phosphorylation processes involved in this phenomenon are associated with transferring the signal to the cell nucleus with the involvement of Smad proteins (intracellular proteins that transduce extracellular signals from TGF-β1 ligands to the nucleus), which act on different transcription factors in the cell nucleus leading to the expression of various genes (48). It should be emphasised that TGF-β, apart from the Smad-dependent signal transduction pathway, may also affect mitogen-activated protein kinase (MAPK) (45,48,51–53). The expression of TGF-β, an important pro-fibrotic signalling molecule, is mediated by a number of factors, including numerous cytokines and chemical agents. However, these processes have not been fully understood in asthma pathogenesis, which is evidenced by limited studies on this subject (30,47,51); the asthma phenotype depends also on other very complex gene-gene and gene-environmental factor interactions.

In conclusion, a complex etiopathogenetic process leading to bronchial hyperresponsiveness, recurrent wheezing episodes, cough and dyspnea is determined by the interactions of genetic and environmental factors.

In the context of the widespread discussion on the significance of genetic factors in asthma, an attempt was made in the present study to evaluate the role of TGF-β1 gene polymorphisms in the pathogenesis of this disease. There have only been limited studies on the clinical importance of TGF-β1 gene SNPs in asthma, whose data was collected on small groups of subjects. In the present study, the frequencies of 3 possibly significant SNPs (C−509T, C+466T and T+869C) of the TGF-β1 gene were evaluated. The TT genotype of only the C−509T SNP was found to be statistically significant and to be clinically associated with the loss of asthma symptom control. The levels of TGF-β1 mRNA expression between the groups studied were also assessed and a higher level of this gene expression in the group of patients, as well as its connection with the duration of asthma were detected. However, it was not possible to identify the role of factors affecting the level of gene expression, such as allergies, drugs, pulmonary function parameters, smoking and asthma exacerbations. Moreover, potential tendencies/associations between the TGF-β1 polymorphism and the mRNA expression and level of asthma symptom control and the severity of the disease were indicated. Due to the small number of subjects studied, these associations require further investigation, particularly multi-center ones involving larger study populations. The potential role of the TT genotype of the C−509T SNO of TGF-β1 in the identification of patients at a higher risk of a severe course of asthma and in planning individual pharmacotherapy is worth emphasizing. This marker may in future become an important diagnostic tool in the hands of clinicians. To sum up briefly, asthma is a multifactorially determined disease and asthmatics form a heterogeneous group of patients.

## Figures and Tables

**Table I:** The results of the epidemiological data and lung function tests.

	Group	
	
Parameter	Control	Asthma
Age (years)		
Means ± SD	45.60±16.55	50.03±15.76
Median (interquartile range)	47 (29–58)	52 (37–62)
Min-Max	19–80	19–82
FEV1 (liters)		
Means ± SD	2.99±0.84	2.18±0.86
Median (interquartile range)	2.94 (2.36–3.60)	2.13 (1.53–2.69)
Min-Max	1.28–5.67	0.37–4.68
FEV1 (%) of predicted value		
Means ± SD	95.07±12.64	72.96±20.33
Median (interquartile range)	94.0 (89.0–101.0)	74.0 (59.3–87.0)
Min-Max	38.0–128.0	19.0–125.0
FEV1/FVC (%)		
Means ± SD	79.50±6.14	66.96±12.24
Median (interquartile range)	78.85 (75.0–83.88)	67.69 (58.35–76.99)
Min-Max	64.15–95.63	33.33–91.03
FVC (liters)		
Means ± SD	3.77±1.07	3.22±1.08
Median (interquartile range)	3.55 (3.02–4.52)	3.07 (2.36–3.90)
Min-Max	1.68–7.51	1.11–6.52
FVC (%) of predicted value		
Means ± SD	100.71±14.82	90.54±17.69
Median (interquartile range)	100.0 (91.0–108.0)	91.0 (79.0–102.0)
Min-Max	38.0–150.0	46.0–134.0

SD, standard deviation; FEV1, forced expiratory volume in 1 sec; FVC, forced vital capacity; Min-Max, minimum-maximum.

**Table II:** Detailed classification of asthma severity.

Classification of asthma based on the level of severity	N	%
Asthma	163	100.00
Not severe	108	62.43
Severe	65	37.57
Mild chronic	29	16.76
Moderate chronic	79	45.66
Severe chronic	65	37.57

N, number; %, percentage.

**Table III:** Detailed primer sequences used for the PCR reaction with the characteristic starter binding temperature for complementary DNA matrix sites.

SNP name	Primer sequence (5′→3′)	Starter binding temperature
C−509T	F: CCGCTTCTGTCCTTTCTAGG R: CAGGCGGAGAAGGCTTAATC	60°C
C+466T	F: GGTGGACCTTGTAACCAGCC R: TCAGAGACTGACTCCACCCC	64°C
T+869C	F: TACCAGATCGCGCCCATCTA R: GATGGCCTCGATGCGCTTC	64°C

F, forward; R, reverse; PCR, polymerase chain reaction.

**Table IV:** Detailed sequences of primers used for the PCR-HRM reaction with their characteristic starter binding temperature for complementary DNA matrix sites (1:50).

SNP name	Primer sequence (5′→3′)	Starter binding temperature
C−509T	F: GTGTCTGCCTCCTGACCCTCC R: GCCTCCGGAGGGTGTCAGTG	62°C
C+466T	F: CATGTCCTCACCTGGTACAGC R: CCTGAACCCGTGTTGCTCTC	64°C
T+869C	F: CTGTTCGCGCTCTCGGCAG R: CCAGTAGCCACAGCAGCGG	68°C

F, forward; R, reverse; PCR-HRM, polymerase chain reaction-high-resolution melting.

**Table V:** The frequency distribution of C−509T (rs1800469) SNP genotypes in the control group and the group of patients with asthma.

Form	Control N (%)	Asthma N (%)	p-value	Agreement of the genotype distributions in the controls with the Hardy-Weinberg law (p-value)	Agreement of the genotype distributions in the patients with the Hardy-Weinberg law (p-value)
TT	72 (44.44)	64 (36.99)			
CT	86 (53.09)	104 (60.12)	0.3814	0.0002	<0.0001
CC	4 (2.47)	5 (2.89)			

N, number; %, percentage.

**Table VI:** The frequency distribution of the C+466T (rs200482214) SNP genotypes in the control group and the group of patients.

C+466T SNP	Control N (%)	Asthma N (%)	Agreement of the genotype distributions in the controls with the Hardy-Weinberg law (p-value)	Agreement of the genotype distributions in the patients with the Hardy-Weinberg law (p-value)
TT	163 (100)	173 (100)		
CT	0 (0)	0 (0)	1	1
CC	0 (0)	0 (0)		

N, number; %, percentage.

**Table VII:** The frequency distribution of the T+869C (rs1800470) SNP genotypes in the control group and the group of patients.

C+466T SNP	Control N (%)	Asthma N (%)	Agreement of the genotype distributions in the controls with the Hardy-Weinberg law (p-value)	Agreement of the genotype distributions in the patients with the Hardy-Weinberg law (p-value)
TT	163 (100)	173 (100)		
CT	0 (0)	0 (0)	1	1
CC	0 (0)	0 (0)		

N, number; %, percentage.

**Table VIII:** The frequency distribution of the C−509T (rs1800469) SNP genotypes in the control group and the group of patients.

	p=0.4190		
	
Genotype	Mild chronic asthma, N (%)	Moderate chronic asthma, N (%)	Severe chronic asthma, N (%)
TT	7 (24.14)	32 (40.51)	25 (38.46)
CT	21 (72.41)	46 (58.23)	37 (56.92)
CC	1 (3.45)	1 (1.27)	3 (4.62)
Total (%)	29 (16.76)	79 (45.66)	65 (37.57)

N, number; %, percentage.

**Table IX:** The degree of control of asthma according to the ACT in groups of patients with mild, moderate and severe levels of illness, against the frequency distribution of C−509T SNP genotypes.

	Severity of asthma								
	
	Mild chronic			Moderate chronic			Severe chronic		
			
Genotype	NK N (%)	CK N (%)	K N (%)	NK N (%)	CK N (%)	K N (%)	NK N (%)	CK N (%)	K N (%)
TT	6 (42.9)	1 (7.1)	0 (0.0)	20 (47.6)	9 (29.0)	3 (50.0)	21 (42.9)	4 (26.7)	0 (0.0)
CT	8 (57.1)	12 (85.7)	0 (0.0)	21 (50.0)	22 (71.0)	3 (50.0)	26 (53.1)	11 (73.3)	0 (0.0)
CC	0 (0.0)	1 (7.1)	0 (0.0)	1 (2.4)	0 (0.0)	0 (0.0)	2 (4.1)	0 (0.0)	1 (100)
N	14 (50.0)	14 (50.0)	0 (0.0)	42 (53.16)	31 (39.24)	6 (7.59)	49 (75.38)	15 (23.08)	1 (1.54)

NK, uncontrolled asthma (ACT <20 points); CK, partially controlled asthma (ACT =20–24 points); K, controlled asthma (ACT =25 points); N, number; %, percentage; ACT, asthma control test.

**Table X:** Comparative analysis for genotypes of the C−509T polymorphism between patients with uncontrolled asthma (ACT <20 points), and those with partially controlled and controlled asthma (ACT ≥20 points).

	Asthma ACT <20 N (%)	Asthma ACT ≥20 N (%)	TT vs. CC/CT	CC vs. CT/TT
TT	47 (44.76)	17 (25.37)	OR 2.38	OR 0.96
CT	55 (52.38)	48 (71.64)	CI 1.22–4.66	CI 0.16–5.86
CC	3 (2.86)	2 (2.99)	p=0.0103	p=1.0000

N, number; %, percentage; ACT, asthma control test; CI, confidence interval; OR, odds ratio.

**Table XI:** Expression level of TGF-β1 in the group of patients and the controls.

Level of expression of TGF-β1	Group	Mean ΔC_T_ TGF-β1 mRNA	Median ΔC_T_ TGF-β1 mRNA	Min. ΔC_T_ TGF-β1 mRNA	Max. ΔC_T_ TGF-β1 mRNA	Lower quartile	Upper quartile	SD
ΔC_T_ TGF-β1 mRNA	Patient	−10.20	−10.29	−18.47	−3.05	−11.90	−8.37	3.17
ΔC_T_ TGF-β1 mRNA	Control	−8.59	−8.84	−14.14	−0.46	−9.92	−6.95	2.67

SD, standard deviation; TGF-β1, transforming growth factor-β1.

**Table XII:** Expression level of TGF-β1 in the patient group with regard to the degree of the severity of the illness.

	p=0.2772		
	
	Mild chronic asthma	Moderate chronic asthma	Severe chronic asthma
Expression level of TGF-β1	−12.25±1.77	−9.63±3.07	−10.15±3.44

SD, standard deviation; TGF-β1, transforming growth factor-β1.
